# The Association between Air Pollution and Outpatient and Inpatient Visits in Shenzhen, China

**DOI:** 10.3390/ijerph15020178

**Published:** 2018-01-23

**Authors:** Yachuan Liu, Shanen Chen, Jian Xu, Xiaojian Liu, Yongsheng Wu, Lin Zhou, Jinquan Cheng, Hanwu Ma, Jing Zheng, Denan Lin, Li Zhang, Lili Chen

**Affiliations:** 1 Department of Statistics, University of California, Berkeley, CA 94720, USA; liuyachuan7119@hotmail.com; 2 Department of Industrial Engineering and Management, Peking University, Beijing 100871, China; shanen.chen@pku.edu.cn; 3 IBM Research China, Beijing 100193, China; xujianx@cn.ibm.com (J.X.); zhanglir@cn.ibm.com (L.Z.); 4 Shenzhen Center for Disease Control and Prevention, Shenzhen 518073, China; xjliu@szcdc.net (X.L.); zhoulin_szcdc@163.com (L.Z.); cjinquan@szcdc.net (J.C.); szmhw@szcdc.net (H.M.); 5 Medical Information Center of Shenzhen Municipality, Shenzhen 518055, China; cnzhengj@163.com (J.Z.); ldn308@163.com (D.L.)

**Keywords:** air pollution, health effects, time series analysis, generalized additive model

## Abstract

Nowadays, air pollution is a severe environmental problem in China. To investigate the effects of ambient air pollution on health, a time series analysis of daily outpatient and inpatient visits in 2015 were conducted in Shenzhen (China). Generalized additive model was employed to analyze associations between six air pollutants (namely SO_2_, CO, NO_2_, O_3_, PM_10_, and PM_2.5_) and daily outpatient and inpatient visits after adjusting confounding meteorological factors, time and day of the week effects. Significant associations between air pollutants and two types of hospital visits were observed. The estimated increase in overall outpatient visits associated with each 10 µg/m^3^ increase in air pollutant concentration ranged from 0.48% (O_3_ at lag 2) to 11.48% (SO_2_ with 2-day moving average); for overall inpatient visits ranged from 0.73% (O_3_ at lag 7) to 17.13% (SO_2_ with 8-day moving average). Our results also suggested a heterogeneity of the health effects across different outcomes and in different populations. The findings in present study indicate that even in Shenzhen, a less polluted area in China, significant associations exist between air pollution and daily number of overall outpatient and inpatient visits.

## 1. Introduction

Ambient air pollution was at least suspected to cause adverse health effects, which was estimated to be responsible for more than 2 million deaths annually and 6.4 years of lost life worldwide [1]. Exposures to air pollution increase risk in mortality, particularly in susceptible populations, and present enormous burden on existing public health system. Assessing the association between air pollution and diseases can allow a better coordination of mitigation and intervention resources.

A broad epidemiological studies have shown that increased ambient air pollutant concentrations are associated with excess daily mortality [2], hospital admissions [3] and emergency hospital visits [4]. These research studies were conducted to assess health impact of air pollution on specific diseases. For instance, associations have been observed between air pollution and emergency department visits for asthma [4,5,6], particularly for effects of nitrogen dioxide (NO_2_), ozone (O_3_), sulfur dioxide (SO_2_) and particulate concentrations. Air pollutants including NO_2_, SO_2_ and particulate matter less than 2.5 µm in aerodynamic diameter (PM_2.5_) have also been reported to be risk factors of hospital admissions for cardiovascular diseases [7], hypertension [8] and respiratory infection [9]. For example, Xia et al. [9] found that acute respiratory infection hospital admission would significantly increase when people exposed to ambient air pollution in Shenzhen in 2013. However, few studies were devoted to pooled estimate of air pollution health effects using overall hospitalized outpatient and inpatient visits. The comprehensive estimate of health effects for air pollution is necessary for us to implement better disease control policy.

Furthermore, most previous studies focused on heavily polluted areas such as Beijing or Shanghai whereas associations between air pollutant concentrations and health effect metrics were less addressed, especially in China. For heavily polluted areas, Guo et al. [10,11] conducted health risk assessment of air pollution on hypertension and cardiovascular diseases in Beijing and Tianjin, respectively, where both cities were of high air pollutant concentrations. Similar studies that highlighted the air pollution health effects in heavily polluted areas could also be found in other literature [12]. On the other hand, relations between pollution and hospital visits for specific diseases have also been reported from areas with low-level air pollution concentration [13,14], although less frequently. Oudin et al. [14] investigated the health effects of daily O_3_, particulate matter less than 10 µm in aerodynamic diameter (PM_10_), and NO_x_ on hospital visits for stroke in Southern Sweden where pollutant concentrations were rather low. Increased risk for ischemic stroke hospital visits at certain level of PM_10_ has been observed, whereas no consistent associations were found for ischemic stroke and O_3_ or NO_x_. Consequently, particularly in China, there is evidence of associations between certain diseases and air pollutions where air pollution concentrations are high, whereas the studies on health effects of air pollutions in less polluted areas are insufficient.

Shenzhen, a less polluted city of China, is a part of the Pearl River Delta region. With the development of industrialization and urbanization, different severe air pollutants, such as PM_10_ and PM_2.5_ [15,16], NO_2_ [17] and O_3_ [18] have appeared in the Pearl River Delta region. The objective of the present study is to explore associations between ambient air pollution and overall hospital outpatient and inpatient visits at locations with comparatively low concentrations of pollution where air quality index is usually less than 100. Data including air pollutant concentrations, meteorological factors and inpatient and outpatient visits were obtained from environmental protection bureau, meteorological bureau, and different hospitals in Shenzhen. The effects of air pollutants, namely SO_2_, carbon monoxide (CO), NO_2_, O_3_, PM_10_ and PM_2.5_ were analyzed with a time series analysis.

## 2. Materials and Methods

### 2.1. Data Collection for the Hospital Outpatient and Inpatient Visit

Situated at a degree south of the Tropic of Cancer, south of China, Shenzhen has a warm, humid subtropical climate, and is less polluted due to geographical location, climatological parameters and low degree of industrialization. To evaluate the health effect of air pollution in Shenzhen, data on daily hospital outpatient and inpatient visits from 1 January 2015 to 31 December 2015 were collected from Center for Disease Control and Prevention (CDC) of Shenzhen. 25,185 records of outpatient and inpatients visits for a total of 69 hospitals in Shenzhen, including a pediatric, an ophthalmological and a cardiovascular hospital, were obtained. Most of general hospitals in Shenzhen are open regularly all day from Monday to Sunday, however several special hospitals could be close on holidays, for which records of zero outpatient and inpatient visits could be observed. To improve the reliability of analysis, 17 hospitals that meet the following standards were removed for further analysis:

- The ratio of zero records was greater or equal to mean of that for all hospitals
- The ratio of outliers (records fall outside mean ±2 standard deviation) was greater or equal to mean ratio of outliers +2 standard deviations
- The coefficient of variation was greater or equal to mean ±2 standard deviations

Records of the remaining 52 hospitals was further filtered with mean ±2 standard deviations criteria.

### 2.2. Data Collection for Air Pollution and Weather Condition

The daily concentrations of ambient air pollutants in 2015 were obtained from Shenzhen Environmental Protection Bureau website. There are 19 fixed monitoring stations in the city, which are distributed in six administrative areas and four new areas in Shenzhen. Daily average concentrations of monitoring stations on SO_2_, CO, NO_2_, O_3_, PM_10_ and PM_2.5_ were included in this study. If data of air pollutants were missed on a given day, the missing values would be calculated with the average concentration computed from the remaining data. Of note, the air pollution data was representative to analyze the exposure of city population. Daily temperature, humidity and barometric pressure data were also collected from Shenzhen Meteorological Bureau website to adjust the effects of air pollution.

### 2.3. Statistical Analysis

In present study, Poisson regression using a generalized additive modeling technique was performed to analyze the associations between ambient air pollutant concentrations and outpatient and inpatient visits. The dependent variables were the overall hospital daily count of outpatient and inpatient visits. In most analyses, the 3-day moving average pollutant concentration (the average of concentrations today (lag 0), yesterday (lag 1), and 2 days ago (lag 2) were modeled [19]. To further describe the associations, this study examined the moving average concentrations from 2 to 8 days (lag 0~1, lag 0~2, ……, lag 0~7). Moreover, air pollutant concentrations at lag time of 1–7 days were tested for significance. The relevant daily data of air temperature (minimum, average and maximum), relative humidity, and barometric pressure were incorporated into the models as confounding factors. Regression models also accounted for time and day of the week with six dummy variables. Additionally, to assess the effect of air pollutants on specific population, this study performed separate analysis on data with regards to general hospitals, pediatric hospital, ophthalmological hospital and cardiovascular hospital.

The anti-log of the regression coefficients for the major pollutants in the generalized additive models is a rate ratio, also interpreted as relative risk (RR). Increased risk (RR−1 * 100%) and the associated 95% confidence intervals (95% CI) for per 10 µg/m^3^ increase in air pollutants were further derived from estimated RRs, as observed during the study period. All statistical tests were two-sided. Only those independent variables with a test values of *p* < 0.05 were considered statistically significant. In addition, all the analysis was performed with R 3.2.3 on a Linux server.

## 3. Results

Table 1 shows the summary statistics of daily hospital visits for 49 hospitals, air pollution, and weather condition of Shenzhen in 2015.

The average daily outpatient visits for 49 hospitals were 100,495 (maximum 122,731, minimum 70,167). The average daily inpatient visits were 2540 with a maximum of 3348 and a minimum of 1756. Air pollutant measurements were available for 365 days, of which the daily average concentrations of SO_2_, CO, NO_2_, O_3_, PM_10_, and PM_2.5_ were 8.33 µg/m^3^, 850.45, 33.36, 55.53, 49.11, and 29.87 µg/m^3^, respectively, wherein all the air pollutants were lower than the national primary ambient air quality standard in China (20 µg/m^3^, 4000 µg/m^3^, 40 µg/m^3^,100 µg/m^3^, 50 µg/m^3^, and 35 µg/m^3^, respectively).

**Figure 1:**
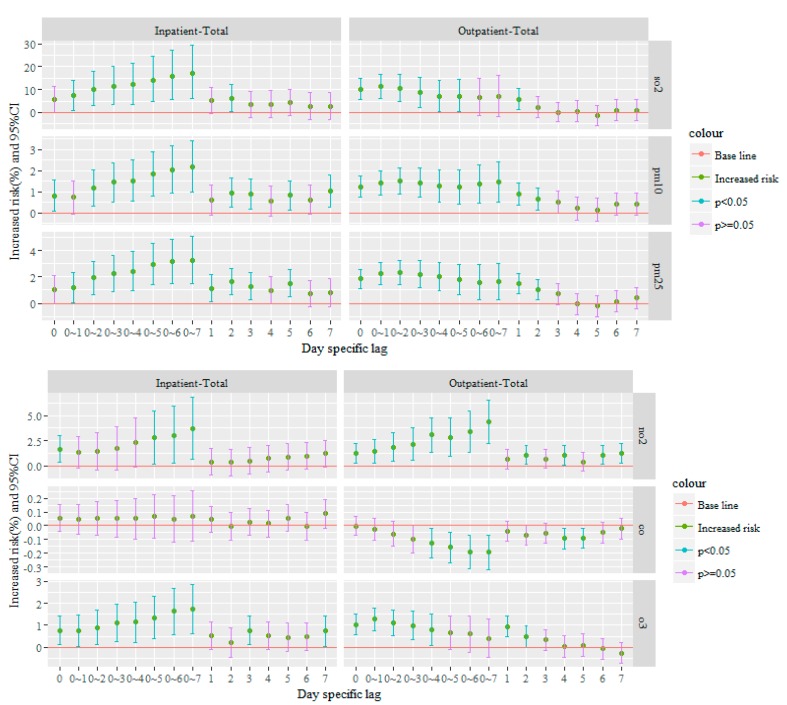
Increased risk estimates and 95% CI between each pollutant and overall outpatient and inpatient visits under different day specific lag associated with a 10 µg/m^3^ increase in air pollutants.

The increased risk and the associated 95% CI for each air pollutant (at different lags) were evaluated by controlling the influence of temperature, relative humidity, pressure and day of the week. Figure 1 displays increased risk for day-specific lags (lags 0–7), and day-specific moving average concentrations from 2 to 8 days (lag 0~1, lag 0~2, ……., lag 0~7) for association between ambient air pollutant concentrations and hospital visits (outpatient and inpatient visits).

The overall association estimate for per 10 µg/m^3^ increase in air pollutants at different lags were also summarized in Table 2. For overall outpatient visits, the highest increases in visits associated with each 10 µg/m^3^ increase in air pollutants were 11.48% (95% CI: 6.32–16.89%) for SO_2_, 4.38% (95% CI: 2.25–6.56%) for NO_2_, 1.28% (95% CI: 0.76–1.80%) for O_3_, 1.52% (95% CI: 0.90–2.13%) for PM_10_, and 2.36% (95% CI: 1.45–3.28%) for PM_2.5_, respectively. Estimates from this distributed lag generalized additive model suggested that there were both immediate and lagged effects for these air pollutants. For all pollutants except CO, the pollutant concentrations on the day of outpatient visits (lag 0) as well as within 5-day moving average concentrations were significantly associated with overall outpatient visits. Interestingly, marginally reduced risk has been observed between CO and overall outpatient visits. As for overall inpatient visits, increased health risk effects were found between air pollutants consisting of SO_2_, NO_2_, O_3_, PM_10_, PM_2.5_ and inpatient visits, with highest increase in visits associated with each 10 µg/m^3^ increase in air pollutants being 17.13% (95% CI: 6.13–29.26%) for SO_2_, 3.66% (95% CI: 0.63–6.77%) for NO_2_, 1.70% (95% CI: 0.59–2.84%) for O_3_, 2.20% (95% CI: 1.01–3.41%) for PM_10_, and 3.27% (95% CI: 1.51–5.07%) for PM_2.5_. Among the above associations, the strongest associations between air pollutants and inpatient visits was found at 8-day moving average pollutant concentration. For the other associations (CO), the effects on hospital inpatient visits were not statistically significant at any lags of CO.

A total of 49 hospitals have been divided into four categories, namely general hospitals, pediatric hospital, ophthalmological hospital and cardiovascular hospital. Generalized additive models have performed separately on data of four categories of hospitals. Thereafter, associations with each 10 µg/m^3^ increase in air pollutant concentration and 95% CI were estimated similarly by controlling temperature, relative humidity, pressure, time and day of the week. This study also calculated increase estimates for day-specific lags (lags 0–7), and day-specific moving average concentrations from 2 to 8 days for different categories of hospitals to assess the health effects of ambient air pollution. For general hospitals, all the pollutants investigated in this study were significantly associated with outpatient visits, with a maximum of 13.65% (95% CI: 5.47–22.46%) increase in visits for each 10 µg/m^3^ increase in 2-day moving average concentration of SO_2_. Meanwhile, air pollutants except CO significantly increased risk of inpatient visits, which confirmed to results of overall inpatient visits. For pediatric hospital, no significant associations have been found between outpatient visits and air pollutants, while the risk estimates of pollutants except O_3_ suggested strong relations between air pollution and inpatient visits. Increase in pediatric hospital inpatient visits associated with each 10 µg/m^3^ increase in air pollutant concentrations ranged from 0.16% (95% CI: 0.005–0.31%, CO with lag 7) to 27.94% (95% CI: 8.44–50.96%, SO_2_ with 8-day moving average). The results also showed that SO_2_, NO_2_, O_3_, PM_10_, and PM_2.5_ displayed significant associations for both outpatient and inpatient visits of ophthalmological hospitals. CO increased risk for outpatient visits but did not significantly affect inpatient visits of ophthalmological hospitals. Analyses of outpatient and inpatient visits for cardiovascular hospital revealed that air pollutants except CO increased risk for outpatient visits, while only NO_2_ were observed to be strongly associated with inpatient visits.

## 4. Discussion

The present study analyzed the hospital visits (outpatient and inpatient visits) data obtained from Shenzhen CDC to investigate associations between major air pollutants and hospital inpatient and outpatient visits over a period of 12 months. Significant associations between air pollutants including SO_2_, NO_2_, O_3_, PM_10_, as well as PM_2.5_, and two types of hospital visits were observed. The estimated increase in overall outpatient visits associated with each 10 µg/m^3^ increase in air pollutant concentrations ranged from 0.48% (O_3_ at lag 2) to 11.48% (SO_2_ with 2-day moving average); for overall inpatient visits ranged from 0.73% (O_3_ at lag 7) to 17.13% (SO_2_ with 8-day moving average). For general and ophthalmological hospitals, the strength of the associations is consistent with estimates of overall outpatient and inpatient visits, while different results have been obtained for pediatric and cardiovascular hospitals.

While ambient air pollution consists of a complex mixture of compounds, the study focused on SO_2_, CO, NO_2_, O_3_, PM_10_, and PM_2.5_. SO_2_ is a traditional pollutant of industrial origin related to the combustion of coal and other fossil fuels. Consistent with the previous findings, our study demonstrated that the daily outpatient and inpatient visits were significantly associated with the concentration of SO_2_ [20,21]. A list of studies provided evidence that hospital admission due to allergic rhinitis [22], asthma [18,23] and acute respiratory diseases [24,25] were related to exposure to SO_2_. Increases in various types of admission would elevate the overall hospital admission, which might explain the significant associations between SO_2_ and overall outpatient and inpatient visits in our study.

Our study results shown that CO only had significant estimated associations with inpatient visits to the pediatric hospital, which partially agreed with the previous studies. Pan et al. [26] found that there were positive associations between CO levels and asthma. Bell et al. [27] shown that short term exposure to ambient CO, even at low ambient CO concentration, was associated with risk of cardiovascular disease hospitalizations. In addition, Fusco et al. [28] have evaluated the associations between CO and most of the respiratory conditions in all ages in Rome. They have found that CO was remained an independent predictor for respiratory admission. On the other hand, Villeneuve et al. [29] found that when CO increased by interquartile range of 5-day moving average, the risk of an asthma emergency department visit for children aged 2–4 would increase by 48%. Hajat et al. [30] identified that CO was significant associated with daily asthma visits and other lower respiratory disease in children. Compared with other age groups, children are more susceptible and they would not be affected by the confounders such as smoking, stress, emotional factors and systemic diseases, which may explain, at least in part, why ambient CO was only associated with inpatient visits in pediatric hospital in our study.

The main sources of ambient NO_2_ emission are coal and oil fired power plants and gasoline powered motor vehicle engines. The results of this study shows that increase of NO_2_ concentrations has a statistically significant association with outpatient and inpatient visits for all categories of hospitals. It is in agreement with studies that focused on hospital visits for specific diseases [31]. In both meta-analysis [32] and spatiotemporal analysis [33], robust associations were found for NO_2_ elevation and asthma admission, which support our findings in this study.

O_3_ is a highly reactive gas which might induce bronchial inflammation, constriction of the airways and decrease lung function. The findings in our study have demonstrated strong associations between O_3_ and outpatient visits of general, ophthalmological and cardiovascular hospitals, while yielding inconsistent results for inpatient visits of pediatric and cardiovascular hospitals. Similarly, the previous studies have not yielded consistent results on associations between O_3_ and certain type of hospital admissions. Exposures to O_3_ have been previously reported to be associated with asthma admission and emergency department visits [33]. In contrast, admission due to asthma were not associated with O_3_ in North America [34]. For respiratory conditions, strong association of 10.00% increase in outpatient visits with per 10 µg/m^3^ increase in O_3_ has been reported for total respiratory disease after controlling for potential confounders [35]. However, daily general practice admission for respiratory conditions were unrelated to O_3_ in Taiwan [36]. The effect of O_3_ might be confounded by different types of admission to hospitals, which could explain the inconsistent results obtained in this study.

PM_10_ and PM_2.5_ are reported to be the major indicators of air pollutants which have direct negative bearings on human health. In our study, PM_10_, and PM_2.5_ were associated with increased likelihood of hospital outpatient and inpatient visits with every 10 µg/m^3^ increase in pollutant concentrations. Both PM_10_ and PM_2.5_ are heterogeneous mixture of small solid or liquid particles with varying compositions in the atmosphere, which would impact the outpatient and inpatient visits in a quite similar pattern. For specific disease risk evaluation, research studies found no consistent results between PM_10_ and asthma admissions [37]. Increase of the average levels of PM_2.5_ has been found to increase the daily outpatient visits in respiratory hospitals (per 10 µg/m^3^ increase = 0.35%, 95% CI: 0.12–1.64%), while PM_10_ was not significantly associated with daily outpatient visits of the same hospital [38]. Pan et al. found levels of PM_10_ were associated with outpatient visits for asthma [27]. Hwang and Chan analyzed the data obtained from clinic records and environmental monitoring stations in Taiwan and reported a significant impact of PM_10_ on outpatient visits [36]. These results were consistent with our findings.

Most of previous studies have focused on air pollutants on specific diseases of certain hospitals. The analysis conducted in this study evaluated the associations between different air pollutants and overall outpatient and inpatient visits. A major strength of the study is the high-quality data obtained from the Shenzhen with a high coverage of all hospitalized outpatient and inpatient visits. Since the database includes various types of visits in multiple hospitals and different air pollutant data that are highly representative, we can rule out the possibility of selection bias. Particularly, since the data were obtained from a historical database and collected sufficient information, recall bias was avoided.

There were still several potential limitations in our study. First, though several potential confounders were adjusted in the generalized additive models, a bunch of possible confounding variables such as seasonal effect, family history, occupational exposures were not included in present study. Second, self-treatment with alternative services was not included. Therefore, the extent of the issue may have been considerably underestimated. Finally, potentially inaccurate data in the records could increase the estimation error in statistical analysis.

## 5. Conclusions

In conclusion, the present study provides evidence that ambient air pollution exerted increased health risk effects on health and increased certain types of hospital outpatient and inpatient visits. These findings reinforce the importance of air pollution controls and disease prevention in less polluted areas, and warn the public about the atmospheric factors that could impact public health. This study also provides insight into the planning of clinical services and emergency contingency response for air pollution exposures.

## Figures and Tables

**Table 1:** Summary statistics of outpatient/inpatient visits, air pollution and weather conditions (*n* = 365).

Variable	Mean	Standard Deviation (SD)	Min	Max
Hospital admission				
Outpatient visits	100,495.42	9752.35	70,167.00	122,731.00
Inpatient visits	2540.69	336.90	1756.00	3348.00
Air pollutants (µg/m^3^)				
SO_2_	8.33	2.45	4.12	21.32
CO	850.45	168.41	551.62	1408.88
NO_2_	33.36	11.50	13.67	101.58
O_3_	55.53	23.56	16.00	162.53
PM_10_	49.11	24.40	13.54	160.48
PM_2.5_	29.87	17.09	7.00	100.71
Weather conditions				
Relative Humidity (%)	71.93	11.05	28.00	93.00
Pressure (hPa)	1005.97	6.45	990.60	1019.30
Temperature (°C)	23.96	5.24	11.90	33.00

**Table 2:** Increased risk and 95% confidence intervals for per 10 µg/m^3^ increase in air pollutants for associations between air pollutants and outpatient and inpatient visits.

Lag (Days)	% Changes (95% CI)—Outpatient Visits for All Hospitals					
	SO_2_	CO	NO_2_	O_3_	PM_10_	PM_2.5_
0	10.21 (5.75~14.86)	0 (−0.07~0.07)	1.21 (0.24~2.19)	1.03 (0.57~1.50)	1.24 (0.73~1.75)	1.85 (1.11~2.59)
1	5.87 (1.4~10.54)	−0.04 (−0.11~0.04)	0.65 (−0.32~1.63)	0.93 (0.46~1.4)	0.90 (0.38~1.42)	1.50 (0.74~2.26)
2	2.31 (−2.06~6.88)	−0.07 (−0.14~0.01)	1.09 (0.14~2.05)	0.48 (0.01~0.95)	0.65 (0.14~1.17)	1.01 (0.26~1.77)
3	−0.08 (−4.19~0.21)	−0.05 (−0.13~0.02)	0.68 (−0.27~1.63)	0.34 (−0.13~0.81)	0.51 (−0.01~1.03)	0.69 (−0.08~1.46)
4	0.59 (−3.88~5.26)	−0.09 (−0.17~0.02)	1.06 (0.07~2.06)	0.04 (−0.45~0.54)	0.20 (−0.33~0.75)	−0.06 (−0.86~0.74)
5	−1.55 (−5.79~2.88)	−0.09 (−0.16~0.02)	0.4 (−0.55~1.35)	0.09 (−0.41~0.60)	0.15 (−0.39~0.69)	−0.22 (−1.02~0.58)
6	0.98 (−3.38~5.54)	−0.05 (−0.12~0.02)	1.09 (0.12~2.07)	−0.07 (−0.53~0.40)	0.4 (−0.12~0.94)	0.12 (−0.67~0.92)
7	0.90 (−3.46~5.47)	−0.02 (−0.09~0.05)	1.23 (0.28~2.2)	−0.26 (−0.72~0.20)	0.41 (−0.11~0.93)	0.4 (−0.39~1.18)
0~1	11.48 (6.32~16.89)	−0.02 (−0.1~0.06)	1.39 (0.23~2.58)	1.28 (0.76~1.80)	1.41 (0.84~1.97)	2.23 (1.41~3.06)
0~2	10.62 (4.87~16.69)	−0.06 (−0.15~0.03)	1.86 (0.46~3.26)	1.11 (0.53~1.71)	1.52 (0.90~2.13)	2.36 (1.45~3.28)
0~3	8.61 (2.34~15.26)	−0.1 (−0.2~0)	2.13 (0.54~3.73)	0.99 (0.34~1.65)	1.43 (0.74~2.12)	2.19 (1.16~3.23)
0~4	7.04 (0.30~14.23)	−0.12 (−0.23~0.02)	3.05 (1.32~4.81)	0.77 (0.07~1.49)	1.28 (0.52~2.04)	2.04 (0.97~3.12)
0~5	7.18 (0.46~14.34)	−0.16 (−0.27~0.04)	2.84 (0.95~4.77)	0.65 (−0.12~1.42)	1.23 (0.41~2.05)	1.81 (0.66~2.97)
0~6	6.56 (−1.35~15.11)	−0.19 (−0.31~0.07)	3.4 (1.37~5.47)	0.61 (−0.22~1.44)	1.38 (0.49~2.28)	1.6 (0.29~2.93)
0~7	6.85 (−1.68~16.12)	−0.19 (−0.32~0.07)	4.38 (2.25~6.56)	0.41 (−0.46~1.28)	1.45 (0.50~2.41)	1.64 (0.24~3.05)
**% Changes (95% CI)—Inpatient Visits for All Hospitals**						
0	5.61 (−0.05~11.59)	0.05 (−0.04~0.15)	1.66 (0.36~2.98)	0.77 (0.10~1.43)	0.80 (0.06~1.54)	1.06 (−0.02~2.14)
1	5.19 (−0.45~11.15)	0.05 (−0.05~0.14)	0.4 (−0.88~1.70)	0.52 (−0.1~1.14)	0.61 (−0.08~1.31)	1.13 (0.12~2.15)
2	6.11 (0.35~12.20)	0 (−0.10~0.10)	0.33 (−1.01~1.68)	0.2 (−0.48~0.89)	0.96 (0.26~1.66)	1.64 (0.62~2.67)
3	3.37 (−2.33~9.41)	0.03 (−0.07~0.13)	0.47 (−0.83~1.79)	0.77 (0.13~1.41)	0.89 (0.18~1.59)	1.26 (0.23~2.30)
4	3.44 (−2.26~9.46)	0.01 (−0.08~0.11)	0.72 (−0.58~2.04)	0.53 (−0.11~1.17)	0.57 (−0.14~1.27)	0.95 (−0.07~1.99)
5	4.26 (−1.43~10.28)	0.06 (−0.04~0.1)	0.87 (−0.42~2.19)	0.45 (−0.18~1.09)	0.83 (0.14~1.52)	1.51 (0.51~2.53)
6	2.61 (−3.01~8.56)	0 (−0.11~0.10)	0.96 (−0.34~2.28)	0.49 (−0.15~1.13)	0.63 (−0.07~1.33)	0.72 (−0.3~1.75)
7	2.59 (−3.09~8.61)	0.09 (−0.02~0.19)	1.2 (−0.13~2.55)	0.73 (0.04~1.43)	1.02 (0.27~1.78)	0.79 (−0.27~1.86)
0~1	7.29 (0.82~14.18)	0.05 (−0.06~0.16)	1.31 (−0.26~2.90)	0.74 (0.05~1.45)	0.75 (−0.04~1.53)	1.19 (0.04~2.34)
0~2	10.22 (3.01~17.92)	0.06 (−0.07~0.18)	1.44 (−0.41~3.34)	0.90 (0.14~1.67)	1.19 (0.34~2.05)	1.93 (0.68~3.19)
0~3	11.48 (3.55~20.01)	0.05 (−0.08~0.19)	1.72 (−0.43~3.93)	1.10 (0.26~1.93)	1.46 (0.54~2.38)	2.26 (0.90~3.63)
0~4	12.3 (3.62~21.7)	0.05 (−0.1~0.2)	2.30 (−0.10~4.76)	1.14 (0.23~2.05)	1.54 (0.54~2.54)	2.42 (0.95~3.91)
0~5	14.24 (4.83~24.49)	0.07 (−0.09~0.23)	2.77 (0.17~5.43)	1.34 (0.37~2.32)	1.83 (0.78~2.90)	2.97 (1.41~4.55)
0~6	15.99 (5.79~27.17)	0.05 (−0.12~0.22)	3.04 (0.24~5.92)	1.63 (0.59~2.67)	2.05 (0.93~3.19)	3.18 (1.53~4.86)
0~7	17.13 (6.13~29.26)	0.07 (−0.11~0.25)	3.66 (0.63~6.77)	1.70 (0.59~2.84)	2.20 (1.01~3.41)	3.27 (1.51~5.07)
